# Association and dissociation of the GlnK–AmtB complex in response to cellular nitrogen status can occur in the absence of GlnK post-translational modification

**DOI:** 10.3389/fmicb.2014.00731

**Published:** 2014-12-23

**Authors:** Martha V. Radchenko, Jeremy Thornton, Mike Merrick

**Affiliations:** Department of Molecular Microbiology, John Innes CentreNorwich, UK

**Keywords:** P_II_ protein, GlnK, post-translational modification, uridylylation, ammonium transport, AmtB

## Abstract

P_II_ proteins are pivotal players in the control of nitrogen metabolism in bacteria and archaea, and are also found in the plastids of plants. P_II_ proteins control the activities of a diverse range of enzymes, transcription factors and membrane transport proteins, and their regulatory effect is achieved by direct interaction with their target. Many, but by no means all, P_II_ proteins are subject to post-translational modification of a residue within the T-loop of the protein. The protein’s modification state is influenced by the cellular nitrogen status and in the past this has been considered to regulate P_II_ activity by controlling interaction with target proteins. However, the fundamental ability of P_II_ proteins to respond to the cellular nitrogen status has been shown to be dependent on binding of key effector molecules, ATP, ADP, and 2-oxoglutarate which brings into question the precise role of post-translational modification. In this study we have used the *Escherichia coli* P_II_ protein GlnK to examine the influence of post-translational modification (uridylylation) on the interaction between GlnK and its cognate target the ammonia channel protein AmtB. We have compared the interaction with AmtB of wild-type GlnK and a variant protein, GlnKTyr51Ala, that cannot be uridylylated. This analysis was carried out both *in vivo* and *in vitro* and showed that association and dissociation of the GlnK–AmtB complex is not dependent on the uridylylation state of GlnK. However, our *in vivo* studies show that post-translational modification of GlnK does influence the dynamics of its interaction with AmtB.

## INTRODUCTION

Proteins of the P_II_ signal transduction superfamily play a major role in coordinating the regulation of nitrogen metabolic processes, and have recently also been implicated in regulation of at least one facet of carbon metabolism (Huergo et al., 2013; Rodrigues et al., 2014). They mediate their effects by protein–protein interaction and their targets include key metabolic and regulatory enzymes, transcription factors, and nutrient transporters. The P_II_ proteins are the most widely distributed signalling proteins in nature, with ubiquitous members among prokaryotes, and representatives among nitrogen-fixing Archaea and in the chloroplasts of eukaryotic phototrophs (Sant’Anna et al., 2009; Huergo et al., 2013). They are highly conserved homotrimeric proteins consisting of 12–13 kDa subunits that form a cylindrical body with three protruding T-loops have been shown to be capable of adopting a variety of conformations (Conroy et al., 2007; Truan et al., 2010; Huergo et al., 2013). In the majority of cases where the structure of a P_II_ protein bound to one of its targets has been determined the T-loops play a key role in that interaction.

The universally conserved mechanism by which P_II_ activity is regulated involves binding of the effector molecules 2-oxoglutarate (2-OG) and Mg-ATP or ADP in the lateral clefts formed between the momomers (Radchenko et al., 2010; Radchenko and Merrick, 2011). When the intracellular nitrogen status is low, and 2-OG levels are high, P_II_ proteins bind 2-OG and Mg-ATP in the lateral clefts and in this condition the T-loops are relatively unstructured. However when the intracellular nitrogen status rises 2-OG levels fall and 2-OG dissociates from the effector binding site. Studies of *E. coli* GlnK indicate that in the absence of 2-OG the bound ATP is subject to P_II_-mediated hydrolysis to ADP (Radchenko et al., 2013). This in turn leads to a rearrangement of particular residues in the GlnK binding pocket, most notably Gln39 and Lys58, and a concomitant change in the T-loop to form a more defined structure such that the apex of the loop projects very markedly above the protein’s surface (Conroy et al., 2007; Truan et al., 2010). Hence the switch from the MgATP, 2-OG form of P_II_ in N-limited conditions to the ADP-bound form in N-sufficient conditions is reflected in a concomitant change in T-loop structure which could potentially be sufficient to regulate the ability of P_II_ proteins to interact with their cognate targets.

Nevertheless, in addition to this conserved mode of regulation, some P_II_ proteins have another regulatory mechanism that involves post-translational modification of the T-loop. The *E. coli* P_II_ proteins, GlnB and GlnK, are subject to uridylylation of the conserved tyrosine residue (Tyr51) in the T-loop by a uridylyltransferase/uridylyl-removing enzyme (UTase/UR or GlnD; Jiang et al., 1998; Atkinson and Ninfa, 1999). In N-limiting conditions UTase activity is predominant and GlnB, and GlnK are uridylylated, whereas in N-sufficient conditions the intracellular glutamine concentration rises, glutamine binds to GlnD and the UR activity deuridylylates the P_II_ proteins. Uridylylation of P_II_ proteins is widespread in the Proteobacteria (Jiang et al., 1998) and a very similar GlnD-mediated adenylylation of P_II_ proteins occurs in some Actinobacteria (Hesketh et al., 2002; Strosser et al., 2004). Furthermore, in some Cyanobacteria P_II_ proteins are subject to phosphorylation of the T-loop (Forchhammer et al., 2004). It is, however, notable that in many organisms P_II_ is not subject to any form of post-translational modification (Huergo et al., 2013) which raises the question of the biological role of this modification in those organisms where it occurs. We have chosen to investigate this in a model P_II_ interaction, namely that of *E. coli* GlnK with the integral membrane ammonia transporter protein AmtB.

The primary function of *E. coli* GlnK is to regulate the activity of AmtB which is a homotrimer with an ammonia conduction channel through each subunit (Thomas et al., 2000; Coutts et al., 2002; Zheng et al., 2004). In *E. coli* an increase in the cellular N status promotes binding of GlnK to AmtB and the crystal structure of the complex shows that GlnK has a molecule of ADP bound to each monomer. GlnK interacts with AmtB almost exclusively via the T-loops the tips of which insert deeply into the cytoplasmic pore exits of AmtB such that residue Arg47 blocks ammonia conduction into the cell (Conroy et al., 2007). Initial studies of GlnK–AmtB interaction noted that complex formation in response to an ammonium shock occurred synchronously with deuridylylation of GlnK (Coutts et al., 2002) suggesting that association/dissociation of the complex was regulated by the post-translational state of GlnK.

The GlnK–AmtB interaction is highly conserved in bacteria and Archaea as reflected by conserved linkage between the structural genes (Thomas et al., 2000). Indeed it has been proposed that regulation of AmtB is the ancestral function of GlnK (Sant’Anna et al., 2009). However the *glnK amtB* operon is found in many organisms where there is no evidence of post-translational modification of P_II_ and it was noted by Coutts et al. (2002) that this suggested that regulation of the complex in those organisms would require an alternative mechanism. In this study we describe experiments to examine this topic and show that association/dissociation of the GlnK–AmtB complex can indeed respond to the cellular N status in the absence of post-translational modification of GlnK.

## MATERIALS AND METHODS

### STRAINS, PLASMIDS, AND GROWTH MEDIA

The strains and plasmids used are listed in **Table 1**. Plasmid pAD2 and its derivatives were expressed in GT1000 and used for His_6_AmtB–GlnK (wild-type or variants) complex purification. Plasmid pJT25 or its derivatives in *E. coli* BL21(DE3)pLysS were used for overexpression of GlnK (wild type or variants). Derivatives of pAD2, plasmids pADR47A, and pADY51A, and derivatives of pJT25, plasmids pJTR47A, and pJTY51A, were generated using the QuickChange Lightning Site-directed Mutagenesis Kit (Agilent Technologies). *E. coli* strains were routinely grown in Luria Bertani medium, and for nitrogen-limited growth M9Gln medium (Javelle et al., 2005) was used. Ampicillin (100 μg ml^-1^), chloramphenicol (30 μg ml^-1^) were included as appropriate.

**Table 1:** Strains and plasmids.

	Relevant genotype/phenotype	Reference
***Escherichia coli* strain**
GT1000	*rbs lacZ*::IS1 *gyrA hutC_K_*Δ*glnKamtB*	Coutts et al. (2002)
BL21(DE3)pLysS	F^-^ ompT gal dcm lon hsdS_B_(r_B_^-^ m_B_^-^) λ(DE3) pLysS(cm^R^)	Studier et al. (1990)
**Plasmid**
pAD2	*glnK His6amtB*	Durand and Merrick (2006)
pADR47A	*glnK_R47A His6amtB*	This work
pADY51A	*glnK_Y51A His6amtB*	This work
pJT25	*glnK*	Radchenko et al. (2010)
pJTR47A	*glnK_R47A*	This work
pJTY51A	*glnK_Y51A*	This work

### PURIFICATION OF His6-AmtB AND GlnK PROTEINS

GlnK–AmtB complexes and Histidine-tagged AmtB (His6-AmtB) were obtained by purifying the AmtB–GlnK complex as described previously (Durand and Merrick, 2006; Radchenko et al., 2010). GlnK wild-type and variants were purified from whole cell extracts of BL21(DE3)pLysS carrying pJT25, pJTR47A, or pJTY51A that were heated to 80°C for 4 min and centrifuged at 30,000 *g* for 30 min to remove debris (Moure et al., 2012). Supernatants were processed by affinity chromatography on a Heparin column (GE Healthcare), using a step gradient of 5, 10, 20, 40, and 100% elution buffer (protein eluted at 20% step). Equilibration buffer was 50 mM Tris, 0.1 M KCl, pH 7.5, and elution buffer was 50 mM Tris, 1.0 M KCl, pH 7.5.

### ANALYSIS OF GlnK–AmtB INTERACTION *IN VITRO*

The ability of the variant GlnK proteins to interact with AmtB was assessed by *in vitro* binding assays as described previously (Radchenko et al., 2010). For assessment of association His6-AmtB (400 μg) was immobilized on a HIS-Select spin column previously equilibrated with buffer A [50 mM Tris-HCl (pH 8.0), 100 mM NaCl, 10% (v/v) glycerol, 0.05% (w/v) *n*-dodecyl-*N,N*-dimethylamine-*N-*oxide (LDAO)] containing 5 mM imidazole. The flow through was collected, and unbound proteins were removed by two washes with the same buffer. Three additional washes were carried out in buffer A containing 5 mM imidazole, 300 μg GlnK protein and various combinations of effectors. The effector concentrations used were reflective of those measured *in vivo* under conditions of N-limitation and N-sufficiency (Radchenko et al., 2010). As a control, buffer A containing 5 mM imidazole, 300 μg GlnK protein and 0.6 M NaCl was used. The molar ratio of AmtB to GlnK was approximately 1:3. A single column was used for each condition tested. Elution of His_6_AmtB or His_6_AmtB–GlnK complex was performed by the addition of buffer A containing 500 mM imidazole. The eluted fractions were analyzed on 15% SDS-PAGE. For assessment of dissociation, complexes of AmtB with bound GlnK (wild-type or variants) were applied to a HIS-Select spin column in the presence of different combinations of effectors and washed and eluted fractions were analyzed by SDS–PAGE.

### *IN VIVO* DETECTION OF GlnK MEMBRANE SEQUESTRATION FOLLOWING AMMONIUM SHOCK

*Escherichia coli* cultures of GT1000 carrying plasmids pAD2, pADR47A, and pADY51A were grown in M9Gln medium at 30°C and subjected to ammonium shock by the addition of NH_4_Cl to a final concentration of 200 μM. Samples were taken prior to ammonium addition and then at 0.5, 1, 2, 3, 5, 10, and 15 mins after addition. Whole cell protein extracts, membrane and cytoplasmic protein fractions were prepared as described previously (Coutts et al., 2002). Membrane fractions were subjected to two high-salt washes (600 mM NaCl) prior to analysis. Protein quantification of the fractions was determined with Coomassie Plus^TM^ protein assay reagent (Thermo Scientific) using an albumin standard (Thermo Scientific).

To determine the cellular location of GlnK membrane protein fractions were separated by 12.5% SDS-PAGE (5–10 μg/lane), analyzed by Western blotting and detected as described elsewhere (Radchenko et al., 2010). The uridylylation state of GlnK was also assessed for each sample by separation of the different GlnK states (UMP_0_–UMP_3_) using native PAGE followed by Western blotting. All experiments were replicated three times.

## RESULTS

In order to investigate the influence of GlnK uridylylation on the interaction between GlnK and AmtB we constructed two T-loop variants for comparison with the wild-type. Residue Tyr51 was changed to Ala thereby generating a variant that would not be subject to uridylylation. As a control residue Arg 47 was also changed to Ala, generating a variant that would still be subject to modification but lacked the charged residue at the tip of the T-loop in the GlnK–AmtB complex. The variants were analyzed in two assays. Firstly the purified GlnK proteins were analyzed *in vitro* for their ability to interact with AmtB in response to the effector molecules ATP, 2-OG, and ADP. Secondly the ability of GlnK wild-type and the variants to be sequestered to the membrane by AmtB was analyzed *in vivo* during a transition from N-limited to N-sufficient conditions.

### ASSOCIATION AND DISSOCIATION OF GlnK–AmtB COMPLEXES *IN VITRO*

The ability of the variant GlnK proteins to interact with AmtB was assessed by *in vitro* binding assays as described previously (Radchenko et al., 2010). For assessment of association His6-AmtB was bound to a HIS-Select spin column and the ability of GlnK wild-type and variants to bind to AmtB in the presence of a variety of effector concentrations was studied. For assessment of dissociation complexes of AmtB with bound GlnK (wild-type or variants) were bound to a HIS-Select spin column and the dissociation of GlnK in the presence of a variety of effector concentrations was studied. The effectors concentrations used were reflective of those measured *in vivo* under conditions of N-limitation and N-sufficiency (Radchenko et al., 2010).

**FIGURE 1:**
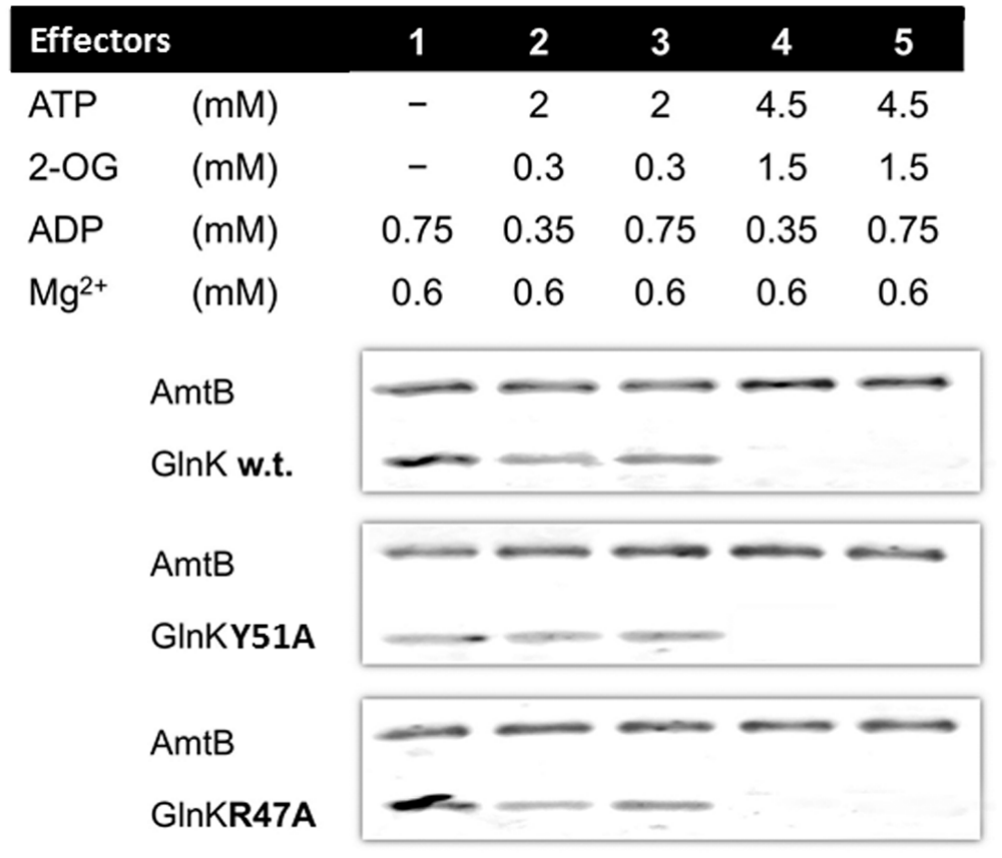
**GlnK–AmtB association *in vitro.*** His6-AmtB was bound to a HIS-Select spin column and GlnK proteins were then applied to the column in the presence of various combinations of effectors. The His6-AmtB or the GlnK–His6AmtB complex were then eluted with 500 mM imidazole and analyzed by SDS-PAGE. The presence of GlnK in the eluted fraction indicates association to AmtB.

**FIGURE 2:**
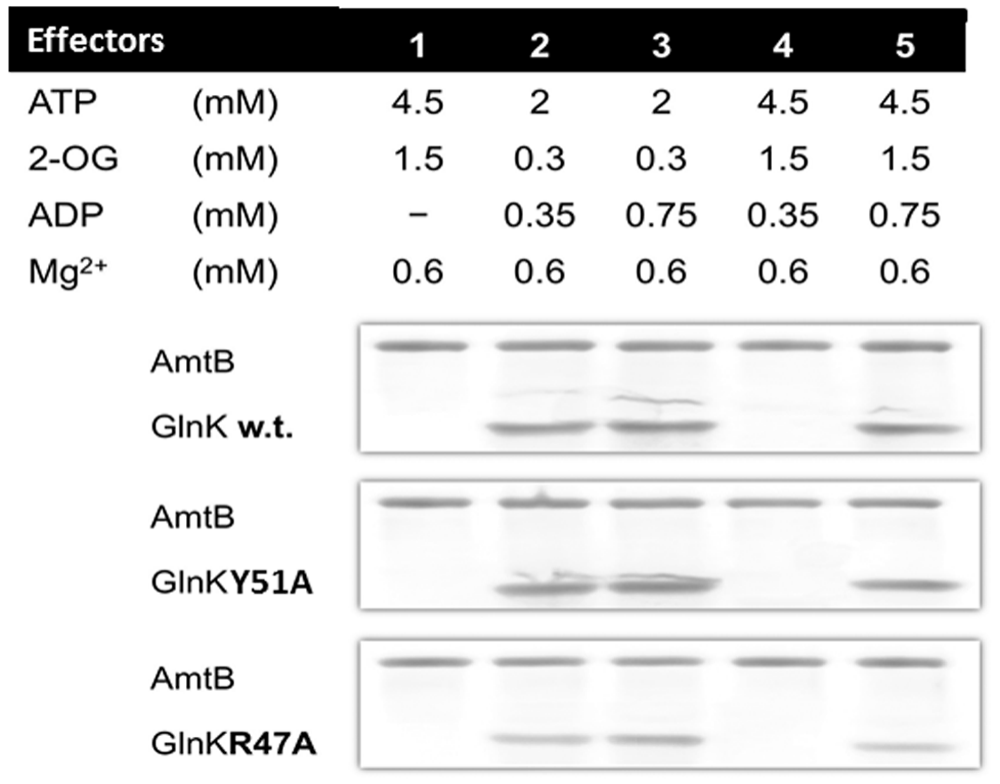
**GlnK–AmtB dissociation *in vitro.*** GlnK–AmtB complexes containing wild type or variant GlnK were bound to a HIS-Select spin column and then subject to washes with buffer containing various combinations of effectors. The His6-AmtB or the GlnK–His6AmtB complex were then eluted with 500 mM imidazole and analyzed by SDS-PAGE. The absence of GlnK in the eluted fraction indicates dissociation of the complex.

In all conditions tested the GlnK variants, Arg47Ala, and Tyr51Ala, behaved in the same way as wild-type GlnK. In N-limited conditions *in vivo* the intracellular 2-OG pool is around 1.5 mM and this falls rapidly following ammonium shock to 0.3 mM (Radchenko et al., 2010). GlnK behavior *in vitro* reflects this, in that association with AmtB requires a low 2-OG level (**Figure 1**). Following ammonium shock intracellular ADP levels rise transiently to around 0.75 mM (Radchenko et al., 2010) and this level of ADP promotes complex formation (**Figure 1**). Dissociation of the complex *in vitro* (**Figure 2**) only occurred in a combination of low ADP (0.35 mM) and high ATP (4.5 mM) and 2-OG (1.5 mM) and these are conditions comparable to those found *in vivo* when cells that are subject to N-limitation (Radchenko et al., 2010).

### GlnK–AmtB COMPLEX FORMATION *IN VIVO*

Cells were grown in N-limitation and then subject to an ammonium shock by addition of NH_4_Cl to the culture medium. Aliquots of the culture were taken prior to ammonium addition and then periodically over a period of 15 mins following the ammonium shock. The interaction of GlnK with AmtB was monitored by detection of GlnK association with the membrane fraction and the uridylylation state of GlnK was monitored by native PAGE as described previously (Radchenko et al., 2010).

**FIGURE 3:**
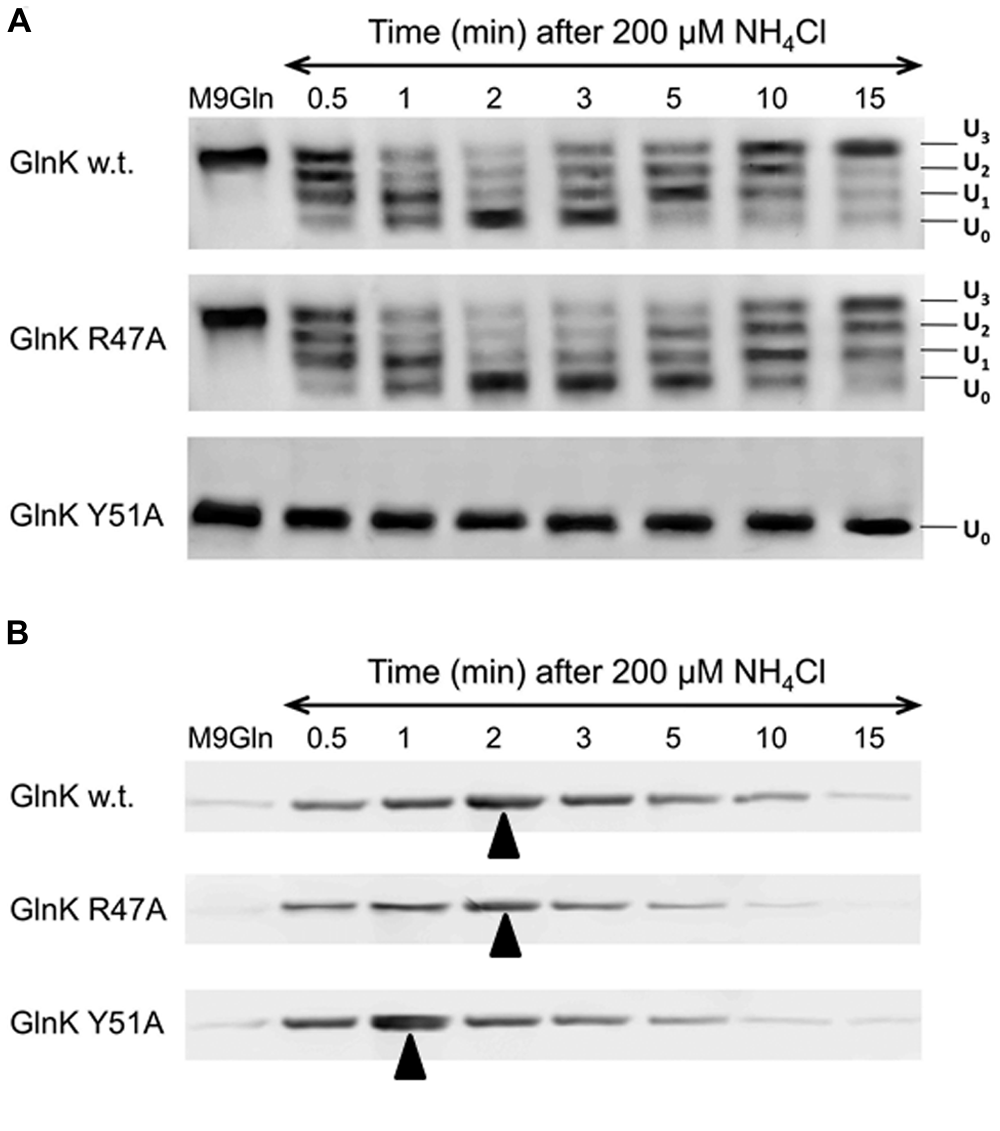
**GlnK–AmtB complex formation *in vivo*. (A)** Changes in the uridylylation state of GlnK wild-type and variants R47A and Y51A following ammonium shock. Culture samples were taken prior to ammonium addition and then at a series of time points and the four possible uridylylation states (UMP_0_–UMP_3_) of the protein were visualized by native PAGE followed by Western blotting. **(B)** Binding of GlnK wild-type and variants R47A and Y51A to AmtB was assessed by Western blotting to detect GlnK association with the cell membrane prior to and following ammonium shock. Arrows indicate point of maximum complex formation.

As reported previously (Radchenko et al., 2010), sequestration of wild-type GlnK to the membrane was maximal 2 mins after ammonium shock and corresponded exactly with the time taken for the protein to become fully deuridylylated (**Figure 3**). As the added ammonium was metabolised the cells returned to N-limitation and 15 mins after ammonium addition GlnK was again dissociated from the membrane and fully uridylylated (**Figure 3**). The GlnK Arg47Ala variant showed an identical membrane sequestration profile to the wild-type. It was also deuridylylated at an identical rate to wild-type but took very slightly longer to be reuridylylated (**Figure 3**). As expected, the Tyr51Ala variant was fully deuridylylated regardless of the N-status of the cells. Nevertheless it still responded to ammonium shock by becoming rapidly sequestered to the membrane. However, in this case the sequestration rate was significantly more rapid than observed with wild-type GlnK or the Arg47Ala variant such that sequestration to the membrane was maximal only 1 min after ammonium shock (**Figure 3**). These experiments were repeated three times with essentially identical results on each occasion.

## DISCUSSION

We have employed a combination of *in vitro* and *in vivo* studies to investigate the role of GlnK uridylylation with respect to the interaction of GlnK with its primary target, the ammonium transport protein AmtB. Prior to the recognition of the role that the effector molecules ATP, ADP, and 2-OG play in influencing the conformation of the T-loop in P_II_ proteins it seemed possible that post-translational modification of the T-loop could be the primary factor controlling the interaction of P_II_ proteins with their targets (Coutts et al., 2002). In such a model, uridylylation of the GlnK T-loop in N-limiting conditions would prevent interaction with AmtB and deuridylylation in response to an elevated cellular N-status would leave the T-loops free to effect complex formation. However, in contradiction of such a model many organisms that encode a *glnK amtB* operon and are predicted to regulate AmtB function by GlnK–AmtB interaction do not have mechanisms for post-translational modification of GlnK.

We have studied two GlnK T-loop variants; one (Tyr51Ala) that is unable to uridylylate GlnK and one (Arg47Ala) that is able to be uridylylated but that lacks the charged residue at the tip of the T-loop which interacts with the pore of AmtB. Both variants behaved *in vitro* in an identical manner to wild-type GlnK in that they showed association with AmtB in the presence of physiological concentrations of ADP (0.35–0.75 mM) excepting in those effector conditions seen in *E. coli* cells subject to N-limitation (4.5 mM ATP, 1.5 mM 2-OG, and 0.35 mM ADP). Hence *in vitro* the Tyr51Ala variant of GlnK was able to associate to and dissociate from AmtB independently of any post-translational modification.

When assessed *in vivo*, for both changes in uridylylation state and dynamics of interaction with AmtB in response to an ammonium shock, the Arg47Ala variant of GlnK behaved essentially as the wild-type. As expected the Tyr51Ala variant was not uridylylated under any of the conditions tested but was fully competent to associate with and dissociate from AmtB in response to the ammonium shock. This confirms that post-translational modification is not essential to regulate the interaction of GlnK with AmtB. Our *in vitro* studies indicate that binding of the effectors, MgATP/ADP, and 2-OG, in response to the metabolic state of the cell is sufficient to control the interaction of GlnK with AmtB through the effect on the conformation of the T-loop. Indeed it seems likely that in many, if not most, cases this could be the primary factor controlling association/dissociation of the P_II_-target complex.

However uridylylation of GlnK did influence the dynamics of GlnK–AmtB association, such that in the absence of the need to deuridylylate the protein GlnK was able to associate more rapidly with its target when the cellular N-status increased. Deuridylylation of GlnK is triggered by a rise in the intracellular glutamine pool and binding of glutamine to GlnD leading to enhancement of its uridyl-removing (UR) activity. Hence it would appear that the deuridylylation of GlnK prior to complex formation with AmtB acts as a check-point that integrates glutamine availability into the signal transduction network. In the proteobacteria and the actinobacteria *glnD* is widespread (Huergo et al., 2013) presumably reflecting a selective advantage of this enhanced signal transduction network. However, in organisms where post-translational modification of P_II_ is apparently absent it seems that regulation of AmtB activity is controlled by the cellular 2-OG pool as the sole measure of N-status. Studies of the P_II_-PipX interaction in *Synechococcus elongatus* have similarly shown that, although P_II_ is subject to phosphorylation of the T-loop, association/dissociation of the P_II_–PipX complex appears to be independent of P_II_ modification (Llacer et al., 2010). Indeed to date there is little evidence of P_II_ target interactions where post-translational modification of the T-loops plays an essential role (Merrick, 2015). There is therefore clearly a need for much more information on the function of post-translational modification of P_II_, both with respect to studies of many more P_II_ interactions with different targets and in a wide range of physiological conditions.

## Conflict of Interest Statement

The authors declare that the research was conducted in the absence of any commercial or financial relationships that could be construed as a potential conflict of interest.
